# Prophylactic effects of probiotics on respiratory viruses including COVID-19: a review

**DOI:** 10.1007/s10068-021-00913-z

**Published:** 2021-05-24

**Authors:** Na-Kyoung Lee, Hyun-Dong Paik

**Affiliations:** Department of Food Science and Biotechnology of Animal Resources, Konkuk University, Seoul, 05029 Republic of Korea

**Keywords:** COVID-19, Probiotics, Prophylactic effect, Gut microbiome, Immunomodulatory effect

## Abstract

Coronavirus disease 2019 (COVID-19), caused by the severe acute respiratory syndrome coronavirus 2 (SARS-CoV-2), is mainly transmitted through respiratory droplets. The symptoms include dry cough, fever, and fatigue; however, high propagation, mutation, and fatality rates have been reported for SARS-CoV-2. This review investigates the structure of SARS-CoV-2, antiviral mechanisms, preventive strategies, and remedies against it. Effective vaccines have been developed by Pfizer (95% effective), AstraZeneca (90% effective), Moderna (94.5% effective) vaccine, among others. However, herd immunity is also required. Probiotics play a major role in the gut health, and some are known to have therapeutic potential against viral infections. Their modes of antiviral activities include direct interaction with targeted viruses, production of antiviral metabolites, and immunomodulatory effects on the host. Hence, probiotics can be a useful prophylactic against COVID-19, and more studies are required on the effects of probiotics against other viral infections that may occur in future.

## Introduction

Coronaviruses are enveloped positive-sense RNA virus with spike (Richman et al., 2016). General coronaviruses can cause mild respiratory diseases. Severe diseases have occurred in humans over the past two decades by the crossover of animal betacorona viruses. First, severe acute respiratory syndrome coronavirus (SARS-CoV) was reported in 2002–2003 in the Guangdong province of China. Then, the Middle East respiratory syndrome coronavirus (MERS-CoV) of bat origin was reported in Saudi Arabia with dromedary camels acting as the intermediate host, infecting 2,492 people and caused 858 deaths (fatality rate 34%) (WHO, 2021). Coronavirus disease 2019 (COVID-19) is caused by the severe syndrome coronavirus 2 (SARS-CoV-2), and originated in China’s Hubei province. COVID-19 was reported in 2019, and recognized as a pandemic on March 11, 2020 by the WHO, eventually spreading in 213 countries, and resulting in more than 120 millions infections and over 2.65 millions deaths worldwide as of March 15, 2021 by WHO.

Probiotics are known to confer health benefits on the host when administered in appropriately amounts (FAO/WHO, 2006), and are used as functional foods for human welfare. Probiotics paly a role in balancing the intestinal microflora and modulating the immune system. Recently, research on probiotics has improved our understanding of the modulation of the gut-liver axis, gut-lung axis, and gut-brain-axis, through the production of IgA and brain-derived neurotrophic factor (BDNF) by the gut microbiome (Vajro et al., 2013). However, probiotics have limitations such as viability control and side effects on hosts; therefore, postbiotics and parabiotics have been investigated as well (Nataraj et al., 2020). Postbiotics are metabolic products of probiotics, such as enzymes, proteins, short chain fatty acids, vitamins, and amino acids, while parabiotics are probiotics inactivated by physical or chemical treatments, which are therefore stable with regard to production and storage, and are considered safer due to lack of side effects such as sepsis.

COVID-19 targets people of all ages, and spreads through large droplets generated during coughing and sneezing by symptomatic patients. SARS-CoV-2 is characterized by high mutation rates. Some patients with COVID-19 show intestinal microbial dysbiosis, and the application of probiotics can balance the intestinal microbiota and reduce the risk of secondary infection (Xu et al., 2020). Therefore, this review deals with the general characteristics of COVID-19 and potential probiotic-related therapeutic strategies for antiviral effects.

### Structure of SARS-CoV-2 and symptoms of COVID-19

SARS-CoV-2 virus is an enveloped, positive sense, single-strand RNA viruses (genome size: 26–32 kb) belonging to the large family of *Coronaviridae* and subfamily *Orthocoronavirinae*, members of which infect birds and mammals (*Coronaviridae* study group of The International Committee on Taxonomy of Viruses, 2020). SARS-CoV-2 binds to angiotensin-converting enzyme 2 (ACE2) receptors on host cells via its spike glycoprotein, which consists of two domains (S1 and S2). S1 binds to the peptidase domain of ACE2, while S2 catalyzes membrane fusion, thereby leading to entry of the viral genetic material into host cell (Hoffmann et al., 2020a, 2020b). The viral RNA codes for structural proteins such as replicase, envelope protein, spike protein, membrane protein, and nucleoprotein; and several non-structural proteins, such as uncharacterized protein 14, and protein 9b (Ou et al., 2020). Non-structural proteins participate in host-protein interactions and modulate host-cell signaling pathways.

The primary infection results from viral transmission through close contact with respiratory droplets from the infected person. The general symptoms of COVID-19 have been reported to be high temperature, dry cough, fever, fatigue, myalgia, and dyspnea (WHO, 2021). Other symptoms include headache, sore throat, rhinorrhea, and gastrointestinal symptoms. The symptoms of COVID-19 manifest after 2–14 days of contact, with latent periods of up to 14 days. The clinical features of COVID-19 vary, and are indistinguishable from other respiratory infections. Adverse outcomes and death are more common among the elderly. Infections in infants and children have been reported to be significantly milder than in adults.

### Antiviral agents and their mechanisms

The RNA genetic material of COVID-19 has ~ 29,811 nucleotides, which encode approximately 29 proteins, which include: structural proteins (4 proteins), nonstructural proteins (16 proteins), and accessory proteins (9 proteins) (Khailany et al., 2020). The four structural proteins are the envelope and membrane proteins that form the viral envelope, nucleocapsid protein that binds to the viral RNA, and the spike S protein that binds to the human ACE2 receptor present on the host cell surface.

Antiviral mechanisms target the life cycle of the virus. The viral life cycle can generally be divided into early- and advanced stages. The therapeutic strategy against COVID-19 has so far involved blocking the early stage of the viral life cycle (Al-Horani et al., 2020), and clathrin-mediated endocytosis (Yang and Shen, 2020).

Quinoline derivatives have been investigated in various settings for the treatment of coronavirus infection (Al-Horani et al., 2020). At the beginning of the COVID-19 pandemic, chloroquine and hydroxychloroquine were authorized for emergency use by the U.S. FDA (U.S. FDA, 2020). Chloroquine was shown to block SARS-CoV-2 infection at a low micromolar concentration in VeroE6 cells (Wang et al., 2020). Chloroquine appears to inhibit the glycosylation of the host ACE2 receptor, which interferes with binding of the virus to the host receptor (U.S. FDA, 2020; Vincent et al., 2005). In addition, chloroquine and hydroxychloroquine have been shown to increase the endosomal/ lysosomal pH, and thus disrupt the viral entry into the host cell (Vincent et al., 2005). However, chloroquine or hydroxychloroquine did not show antiviral effects in patients with COVID-19 (U.S. FDA, 2020).

Antiviral drugs, such as ribavirin and lopinavir-ritonavir, have been used for SARS and MERS. Serious adverse effects have been reported with the use of quinoline-based antimalarial dugs, and chloroquine has been linked to cardiac arrhythmias and retinopathy. Arbidol (an antiviral drug available in Russia and China), plant extracts, intravenous immunoglobulin, interferons, chloroquine, and convalescent plasma, have been investigated for their antiviral effects (Ahn et al., 2020; Bae et al., 2019; Jin et al., 2020; Zhang et al., 2020). Polyphenols have also been used for the prevention of COVID-19 in view of their immune-boosting properties (Mehany et al., 2021). For antiviral therapy, tocilizumab has been used against COVID-19 with/without corticosteroids in February 2021 (NIH, 2019). It is recommended that tocilizumab be administered in combination with dexamethasone. In addition, hospitalized patients were treated for hypoxemia with remdesivir, dexamethasone plus remdesivir, or dexamethasone; and immunotherapy involved administration of corticosteroids, interleukin (IL)-1 or IL-6 inhibitors.

Vaccines are in demand for herd immune, and herd immune might be achieved before 2022. Some vaccines have been permitted for use (BBC News, 2021). Pfizer/BioNtech vaccine has up to 95% effective; however, the vaccine must be stored at a temperature of approximately − 70 °C. The Oxford University/AstraZeneca vaccine has 70–90% efficacy. And the data also showed a strong immune response in the elderly. This may be one of the easiest vaccines to distribute because it does not need to be stored at very cold temperatures. The Moderna vaccine has 94.5% efficacy. It is easier to store than the Pfizer’s vaccine because it remains stable at − 20 °C for up to six months. The Russian Sputnik V vaccine has 92% efficacy. The Wuhan Institute of Biological Products and Sinopharm in China, and Russia's Gamaleya Research Institute, are all in the final testing stages of their respective vaccines. Sinovac (China) has shown 50.4% efficacy in Brazil. Although vaccines are prophylactics against COVID-19, their efficacy is limited by multiple mutations in the virus, such as those giving rise to the UK and South Africa strains, and by their side effects (headache, fever, muscle ache, etc.). In addition, these vaccines are reported to cause severe allergic reactions after vaccine injection in some people.

### Prophylactic therapy by probiotics

The human gut microbiome consists of approximately 1000 different species of microbes, with densities of 10^4^–10^5^ CFU/mL of the digestive tract in the small intestine and 10^11^ CFU/g of luminal content of the colon (Kastl et al., 2020). The gut microbiome principally comprises of four groups, namely *Firmicutes*, *Bacteriodetes*, *Proteobacteria*, and *Actinobacteria* (Sweeney and Morton, 2013). The gut microbiome can help fight infection by competing against pathogens, colonization ability, and metabolite production (e.g., organic acids, antimicrobial compounds, and short chain fatty acids) (Jang et al., 2019).

The gut microbiome influences the health of the host, its imbalance being involved in many conditions such as lung disorders, including asthma, chronic obstructive pulmonary disease, chronic bronchitis, emphysema, lung cancer, pneumonia, pleural effusion, viral infection, and infection (Han et al., 2007). It is also recognized that viral infections in the respiratory tract cause disturbances in the gut microbiome, which points to the existence of the gut-lung axis (Dhar and Mohanty, 2020). Changes in the gut microbiome due to respiratory viral infections have been demonstrated using microbiome analysis and metabolome analysis (Groves et al., 2020). Table 1 showed the relatedness of the viral infection and the gut microbiome. Viral infection results in an increase in the *Bacteriodetes*/*Firmicutes* ratio, and a decrease in microbial diversity in the gut microbiome (Grayson et al., 2018). These changes in gut microbiome result in anorexia by increasing the levels of sphingolipids, polyunsaturated fatty acids (PUFAs), and short-chin fatty acid (SCFA) valerate (Scencio et al., 2020; Yu et al., 2019a). In addition, communication between microbiota and environmental factors affects mucosal immunity (Neish and Jones, 2014). The mucus layer present on the surface of the gastrointestinal tract, respiratory tract, and vaginal tract is the first line of defense, where immunoglobulin A antibody acts as the first line of mucosal immunity (Corthesy, 2013).

**Table 1:** Findings of recent investigations of the activity of gut microbiome against influenza virus

Virus	Findings	References
Influenza virus	Increase in IFN-I levels in lung/production of desaminotyrosine by gut microbe *Clostridium orbiscindens*	Steed et al. (2017)
Influenza virus (H1N1)	Gut microbiome increased anti-inflammatory cytokine (IL-10 and IL-14) levels in H1N1 infection	Rosshart et al. (2017)
Sendai virus	The use of streptomycin in viral infection led to reduction of intestinal microbial diversity These variations influence the increase in mortality and immune responses at distant mucosal sites (decrease in Treg population/increase in IFN-γ, IL-6, and CCL1 levels)	Grayson et al. (2018)
Influenza virus (H1N1/H3N2)	Decrease in short chain fatty acid concentration as dysbiosis	Scencio et al. (2020)

*CCL* CC chemokine ligand, *IFN* interferon, *IL* interleukin

Probiotics have been defined as live microbes that confer health benefits on the host when administered in appropriately amounts (FAO/WHO, 2006; Lee et al., 2019). Probiotics can balance the intestinal microflora, which are involved in the epithelial barrier of intestines, competition with disease-causing agents, attachment to the epithelial wall of intestines, production of anti-pathogen elements, and enhancement of the immune system of the host (Abdulamir and Hafidh, 2020; Jang et al., 2019; Kariyasawasam et al., 2020). *Bifidobacterium bifidum* and *Streptococcus thermophilus* showed a decline in diarrheal incidents and reduction of rotavirus titers in a meta-analysis (Szajewska et al., 2019). The maintenance of commensal bacteria can influence immune homeostasis during invasion by influenza A virus and coronavirus through the gastrointestinal tract. Some probiotics and parabiotics have been reported to reduce the titers of influenza virus, although the underlying mechanism remains to be understood (Park et al., 2018).

**Fig. 1:**
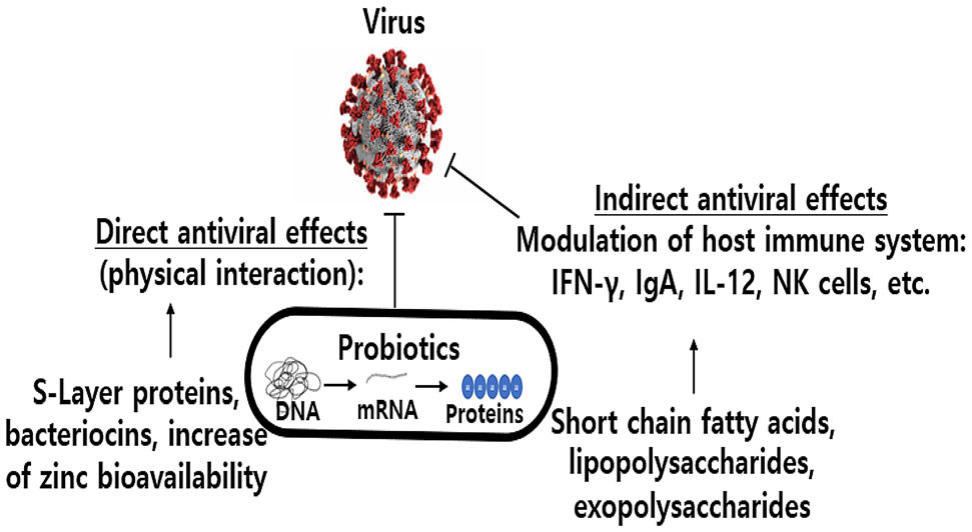
Mechanisms of action of antiviral probiotics against respiratory viruses. Abbreviation: *IFN* interferon, *Ig* immunoglobulin, *IL* interleukin, *NK* natural killer

Probiotics and their metabolites help maintain commensal microbiota despite viral infection (H1N1 and PR8 virus) (Ichinohe et al., 2011). Figure 1 shows the antiviral potentials of probiotics and their metabolites through direct or indirectly interfere with the viral life cycle (Al Kassaa et al., 2014). They exert antiviral activity by direct probiotic–virus interaction, production of antiviral inhibitory metabolites, and indirect modulation of the immune system. Lactic acid bacteria (LAB) and their bacteriocins can serve as antiviral agents (Al Kassaa et al., 2014). LAB are known to synthesize exopolysaccharides, which may confer health benefits to humans, including through immunomodulatory, antitumor, antibiofilm, and antioxidant activities (Jang et al., 2020). In addition, some probiotics, such as *Lactobacillus fermentum* CECT5716, have been found to enhance the effects of influenza vaccination by inducing an antibody response (Olivares et al., 2007).

### Direct probiotic–virus interaction

Some probiotics or their metabolites have been reported to directly inhibit human immunodeficiency virus (HIV) (D’Angelo et al., 2017) and transmissible gastroenteritis virus (TGEV) (Chai et al., 2013). However, the direct inhibition of the influenza virus was limited. *Enterococcus faecium* NCIMB 10415 inhibits influenza virus (H1N1 and H3N2) via direct physical interaction (Wang et al., 2013). The S-layer protein of *Lactobacillus acidophilus* ATCC 4356 inhibits the invasion by and replication of the H9N2 virus (Gao et al., 2016).

In the vesicular stomatitis virus (VSV)–cell culture model, *Bifidobacterium longum* Q46, *Lactobacillus paracasei* A14, and *Lactobacillus plantarum* M1.1 have demonstrated antiviral activity (Botic et al., 2007). Their possible mechanisms include: (1) hindering the adsorption of the VSV, (2) inhibition of virus protection, and (3) production of metabolites with a direct antiviral effect.

Probiotic metabolites include organic acids, bacteriocins, hydrogen peroxide, and its metabolites, which play a role in the health of gut microbiome and host immunity (Tiwari et al., 2020). These substances can inactivate human immunodeficiency virus type I (HIV-1) and herpes simplex virus type 2 (HSV-2) (Conti et al., 2009; Tuyama et al., 2006).

Bacteriocins are ribosomally synthesized antimicrobial peptides with bactericidal activity (Lee et al., 2013). Table 2 shows the antiviral effects of bacteriocins. Enterocin ST4V and CRL35 inhibit HSV-1 or HSV-2 and target the multiplication of viral particles (Todorov et al., 2005; Wachsman et al., 1999a, 1999b). A bacteriocin originating from *Lactobacillus delbrueckii* subsp. *bulgaricus* 1043 inhibits influenza virus (H7N1 and H7N7) (Serkedjieva et al., 2000). Until now, the mechanism underlying the antiviral effects of bacteriocins have not been uncovered; therefore, more research is necessary in this field.

**Table 2:** Antiviral effects and mode of action of some bacteriocins

Bacteriocin (producer)	Virus	Mode of action	References
Bacteriocin (*Lactobacillus delbrueckii*)	Influenza virus (H7N7, H7N1)	Inhibition of invasion and replication of virus	Serkedjieva et al. (2000)
Peptide ST4V (*Enterococcus mundtii* ST4V)	Herpes simplex viruses-1, polio virus (PV3, strain Sabin), and a measles virus (MV/BRAZIL/001/91)	Aggregation of the viral particles or blocking of their receptor sites	Todorov et al. (2005)
Enterocin CRL35 (*Enterococcus mundtii* CRL35)	Herpesvirus	Inhibition of viral replication	Wachsman et al. (1999a, 1999b)
Enterocin ST5Ha (*Enterococcus faecium* ST5Ha)	Herpesvirus-1	Aggregation of viral particles, blockage of receptor sites on the host cell	Todorov et al. (2010)
Enterocin CRL35 (*Enterococcus mundtii* CRL35)	Herpesvirus (HSV-1, HSV-2)	Inhibition of viral replication	Wachsman et al. (2003)
Subtilosine KATMIRA 1933 (*Bacillus amyloliquefaciens* KATMIRA 1933)	Herpesvirus-1	In high concentrations, inhibition of viral particle formation and release	Torres et al. (2013)

### Increase of zinc bioavailability

Zinc plays an important role in membrane integrity, DNA synthesis, and cell proliferation (Read et al., 2019). In addition, zinc is associated with the improvement of the host’s reaction to various infections and plays a significant role in maintaining host homeostasis (Read et al., 2019). Zinc supplementation resulted in a vital reduction in sickness in children with pneumonia (Rerksuppaphol and Rerksuppaphol, 2019).

*Lactobacillus fermentum* SR4 and *Lactobacillus rhamnosus* GG have been reported to increase zinc bioavailability in intestinal cells (Lule et al., 2020). These two strains can chelate zinc at high ratios of 57.9% and 48.2%, respectively, when compared with the commercial chelate (Zn-sulfate (16.1%) and Zn-gluconate (26.9%)).

### Modulation of immune system

Probiotics can influence immunity through various cytokines produced by dendritic cells (DCs), monocytes or macrophages, and B and T lymphocytes (Kawashima et al., 2011). Probiotics are effective against several ailments, including viral infections (Kanauchi et al., 2018). The numbers and the activity of natural killer (NK) cells are also significantly improved by IL-2 activation (Grudzien and Rapak, 2018; Takeda et al., 2006). Innate immunity can benefit from acquired immunity, which is mediated by Toll-like receptors (TLRs) (Belkaid and Hand, 2014). Probiotics can also induce the production of antigen-presenting cell (APC)-derived cytokines (IL-10, IL-12, IL-17, tumor necrosis factor (TNF)-α, etc.) through activation of adaptive immunity. Proinflammatory cytokines, chemokines, and their receptors are inhibited by the anti-inflammatory cytokine IL-10, produced by various immune-activated cells (Azad et al., 2018). As a result, probiotics can have two different types of immunomodulatory effects on inflammation: the immunostimulatory effect, which activates IL-12 production, and induces NK, Th1, and Th2 cells; and immunoregulatory effect, which induces IL-10 and T_reg_ cell activation by Th2, DCs, B cells, and monocytes, through overexpression of cytokines (Chiba et al., 2010).

In some cases, COVID-19 can cause extreme storms of inflammatory cytokines, including IL-2, IL-17, IL-10, granulocyte colony-stimulating factor (GCSF), interferon gamma-inducible protein (IP)-10, monocyte chemoattractant protein (MCP)-1, macrophage inflammatory protein (MIP)1-α, and TNF-α (Chen et al., 2020). Table 3 lists the various antiviral probiotics and their probable mechanisms. *L. plantarum* CRL1506 has demonstrated antiviral properties through modulation of the intestinal immune response (Mizuno et al., 2020). The major antiviral active factor of *L. plantarum* CRL1506 may be lipoteichoic acid (LTA). *L. plantarum* YU has been shown to exert antiviral effects against H1N1 virus by inducing IL-2 secretion (Kawashima et al., 2011). Heat-killed *L. plantarum* L-138 exerts antiviral immunomodulatory effects by inducing interferon (IFN)-β (Maeda et al., 2009). *L. plantarum* 200655 and *Lactobacillus paraplantarum* SC61 have demonstrated immune-enhancing effects (Son et al., 2018; Yang et al., 2019). As stated above, the active factor behind these immunomodulatory effects of heat-killed probiotics might be LTA, a surface glycolipid found in gram-positive bacteria. Some probiotics, such as *L. plantarum* KU15149 and *Weissella cibaria* JW15 exert anti-inflammatory effects by LPS stimulation (Han et al., 2020; Yu et al., 2019b).

**Table 3:** Antiviral effects and mode of action of some probiotics against respiratory viruses

Probiotics	Virus	Mode of action	References
*Enterococcus faecium* NCIMB 10415	Swine influenza virus (H1N1, H3N2)	Direct physical interaction/Strengthening of innate defenses	Wang et al. (2013)
*Lactobacillus acidophilus* ATCC 4356 S-layer protein	H9N2	Inhibition of invasion by and replication of virus; stimulation of type I IFN signaling pathway	Gao et al. (2016)
*Lactobacillus casei* Shirota	Influenza viruses, RV	Activation of immature immune system: activation of NK cells and IL-12 production	Yasui et al. (2004)
*Lactobacillus plantarum* YU	Influenza virus (H1N1)	Activation of Th1 immune response/IgA production	Kawashima et al. (2011)
Heat-killed *Lactobacillus plantarum* L-137	Influenza virus (H1N1)	Immunomodulation; IFN-β induction	Maeda et al. (2009)
*Lactobacillus fermentum* CECT5716/*Lactobacillus casei* DN114-001	Influenza virus	Increase in the antibody response	Boge et al. (2009) and Olivares et al. (2007)
*Lactobacillus rhamnosus* CRL1505	Respiratory syncytial virus	Production of IFN-c and ILs	Villena et al. (2011)
*Lactobacillus rhamnosus* GG/*Bifidobacterium animalis* subsp. *lactis* BB-12/ *Lactobacillus acidophilus* NCFM/ *Bifidobacterium animalis* subsp. *lactis* BI-07/heat killed *Lactobacillus pentosus* b240	Respiratory virus infections	Immunomodulation	Kiso et al. (2013), Leyer et al. (2009), and Rautava et al. (2009)
*Lactobacillus plantarum* NCIMB 8826	Respiratory syncytial virus, pneumovirus	TLR-dependent inflammatory response	Al Kassaa et al. (2014)
*Lactobacillus casei* DN-114001	Respiratory tract infection, rhinopharyngitis, influenza	Enhancement of defensin expression and innate immunity	Guillemard et al. (2010)
*Lactobacillus rhamnosus* M21	Pneumonia, influenza	Increase in IFN-γ and IL-2 levels	Song et al. (2016)
*Lactococcus lactis* JCM 5805	Respiratory tract infection	Activation of plasmacytoid dendritic cells	Kokubo et al. (2019)
*Lactobacillus fermentum* CECT5716/*Lactobacillus plantarum* DK119	Influenza virus	Activation of innate immunity: increase in IL-12, IFN-γ; decrease in IL-4, IL-6, and TNF-α levels	Olivares et al. (2007) and Park et al. (2013)

*IFN* interferon, *Ig* immunoglobulin, *IL* interleukin, *NK* natural killer, *TNF* tumor necrosis factor, *TLR* toll-like receptor

In conclusion, this review investigates the use of probiotics as prophylactics or treatment aids for therapy against COVID-19. Outbreaks caused by zoonotic coronaviruses can result in severe human casualties. Viral infection can lead to gut dysbiosis according to several reviewers. Some probiotics can regulate host homeostasis through immunomodulatory effects and the maintenance of the gut microbiome. However, the use of probiotics is limited as a therapeutic agent. Probiotics have also been shown to reduce the side effects of chemotherapy. The species and mechanisms behind antiviral probiotics vary. Therefore, probiotics can be used as prophylactic medicinal foods against viral infection. Further study is therefore required, to understand the beneficial effects of probiotics against viral infections and their potential use in antiviral therapy.
